# Adult Congenital Heart Disease in the Emergency Department

**DOI:** 10.3390/jpm14010066

**Published:** 2024-01-04

**Authors:** Rachel A. Lindor, Kim Heller, Nicole R. Hodgson, Patrick Kishi, Jessica Monas, Douglas Rappaport, Aaron Thomas, Andrej Urumov, Laura E. Walker, David S. Majdalany

**Affiliations:** 1 Mayo Clinic Department of Emergency Medicine, Phoenix, AZ 85054, USA; 2 Mayo Clinic Department of Emergency Medicine, Rochester, MN 55905, USA; 3 Mayo Clinic Department of Cardiovascular Diseases, Phoenix, AZ 85054, USA; majdalany.david@mayo.edu

**Keywords:** adult congenital heart disease, emergency department

## Abstract

While congenital heart disease historically was a pathology primarily restricted to specialized pediatric centers, advances in technology have dramatically increased the number of people living into adulthood, the number of complications faced by these patients, and the number of patients visiting non-specialized emergency departments for these concerns. Clinicians need to be aware of the issues specific to patients’ individual congenital defects but also have an understanding of how typical cardiac pathology may manifest in this special group of patients. This manuscript attempts to provide an overview of this diverse but increasingly common group of adult patients with congenital heart diseases, including a review of their anatomical variants, the complications they face at the highest rates, and ways that emergency physicians may need to manage these patients differently to avoid causing harm.

## 1. Introduction

Congenital heart disease (CHD) is the most common of all congenital malformations, and as advances in treatment allow more patients to live into adulthood, emergency physicians will continue to encounter these patients with increasing frequency. In this article, we discuss the eight most common forms of CHD, which together comprise 75% of all congenital heart defects, including ventricular septal defects, atrial septal defects, patent ductus arteriosus, pulmonic stenosis, tetralogy of Fallot, coarctation of the aorta, transposition of the great vessels, and aortic stenosis [1]. We briefly review the anatomy and physiology of these defects with a larger focus on the most common clinical presentations of the patients living with them. The goal of this review is to increase emergency physicians’ comfort in recognizing and treating this increasingly prevalent group of patients, who represent a relatively high-risk subset within the emergency department (ED).

## 2. Discussion

### 2.1. Ventricular Septal Defect

#### 2.1.1. Anatomy and Physiology

Ventricular septal defects (VSDs) are the most common form of congenital cardiac malformations, accounting for around 1 in 4 patients born with these [2]. VSDs are characterized by a persistent opening of the interventricular septum. Initially during the neonatal period, as pulmonary vascular resistance remains high, very little shunting occurs. As PVR decreases, left to right shunting occurs resulting in increases in right ventricular and pulmonary vascular pressures. If VSDs do not close, pulmonary hypertension may occur if there is a significant shunt [3]. These may be appreciated upon a physical exam as blowing, holosystolic murmurs best heard close to the sternum, differentiating them from the murmurs of mitral valve regurgitation, which are best heard near the apex with radiation to the axilla.

#### 2.1.2. Medical/Surgical Management

The vast majority of VSDs are asymptomatic given they are small and close spontaneously shortly after birth. As such, these are generally managed conservatively. Medical management typically involves diuretics to treat the symptoms of heart failure that can develop [4]. Common indications for surgery among adults include signs of left-sided volume overload, and significant shunt in the absence of pulmonary hypertension [5].

#### 2.1.3. Special Considerations

Patients with unrepaired VSDs initially have left to right shunts, but as their pulmonary vascular resistance increases, they can have reversal of the shunt known as Eisenmenger syndrome (ES). This right to left shunting would lead to cyanosis with multisystem sequelae such as reduced functional capacity, arrhythmias, and secondary erythrocytosis. All patients with VSDs are at risk for endocarditis [2]. Certain VSDs may be amenable to transcatheter repair although a surgical approach is often required depending on the size and location of the defect [4].

### 2.2. Atrial Septal Defect

#### 2.2.1. Anatomy and Physiology

Atrial septal defects (ASDs) are the second-most common congenital cardiac malformation [1]. ASDs are characterized by a persistent opening between atria, which allows the shunting of blood from the higher pressure left atrium to the lower pressure right atrium. The majority of ASDs occur sporadically, although familial transmission has been described and disease-causing genes identified [6,7,8]. Clinical manifestations of ASD can range from asymptomatic to significant hemodynamic compromise and most patients become symptomatic prior to age 40. Symptoms may include fatigue, exertional dyspnea, exercise intolerance, palpitations, or paradoxical embolism. Patients with large unrepaired ASDs are at risk of developing Eisenmenger syndrome with shunt reversal and cyanosis—occurring in 5–10% of patients. Right-sided volume overload can lead to right atrial and ventricular enlargement, pulmonary hypertension, and potentially right heart failure. Patients with ASDs are at increased risk for atrial fibrillation and flutter, infective endocarditis, as well as recurrent pneumonia [9,10,11].

There are four types of ASD which vary based on location and associated abnormalities. Primum ASD which occurs on the inferior aspect of the inter-atrial septum is associated with cleft mitral valve and easily auscultatable holosystolic murmur of mitral regurgitation. Moreover, compared to other ASDs, primum ASDs have left axis deviation on ECG.

#### 2.2.2. Medical/Surgical Management

Timely intervention is crucial to prevent irreversible pulmonary vascular changes and optimize long-term outcomes. Echocardiography is the imaging modality of choice to evaluate for ASD. The management of ASDs depends on patient characteristics, the size and location of the defect, and symptomatology. Spontaneous closure during infancy or early childhood may occur with small ASDs; however, larger defects often require intervention to prevent further hemodynamic compromise [12]. Some patients are able to undergo transcatheter closure, while those with more complicated defects require surgical repair [5].

#### 2.2.3. Special Considerations

Just as in VSDs, left to right shunting through a large ASD can cause pulmonary hypertension and reversal of the shunt, as in ES. In these patients, ASD closure is contraindicated, as it can worsen right ventricular failure. In patients with increasing pulmonary vascular resistance, medical therapies may be used to counteract this complication [5].

Special consideration should also be given to pregnant women with ASDs due to the increase in cardiac output and decrease in systemic vascular resistance. Women with uncomplicated ASDs generally tolerate pregnancy well, but a history of prior arrhythmia or heart failure can increase risks. Given the hypercoagulable state of pregnancy, paradoxical embolization from deep venous thrombosis is also a concern, and DVT preventive measures, low-dose aspirin after first trimester, or empiric anticoagulation of higher risk patients may be considered [13].

### 2.3. Patent Ductus Arteriosus

#### 2.3.1. Anatomy and Physiology

A patent ductus arteriosus (PDA) is the persistent communication between the left pulmonary artery and the proximal descending aorta. Shunting through the PDA from the aorta back to the pulmonary artery causes increased pulmonary flow and left heart volume overload. The clinical significance of a PDA depends on the size, configuration, and magnitude of the shunt [14]. Clinical presentations range from asymptomatic to pulmonary hypertension (PH), congestive heart failure, endocarditis, atrial fibrillation, and recurrent pneumonia [15].

#### 2.3.2. Medical/Surgical Management

Medical management for adults with symptomatic PDA is short-term until transcatheter closure or surgery can be performed [14]. Transcatheter occlusion of the patent ductus is the preferred treatment. For patients who have developed severe pulmonary arterial hypertension (PAH) and/or Eisenmenger physiology (hypoxemia, central cyanosis), closure is not recommended due to the risk of worsening symptoms [15,16]. Maintaining euvolemic status is important as they can decompensate quickly due to both aggressive fluid resuscitation or diuresis. 

#### 2.3.3. Special Considerations

Patients with pulmonary hypertension who present to the ED have complicated pathophysiology and may be difficult to manage. Careful assessment of their volume status is important with the goal of achieving euvolemia. If patients have an established PAH therapy with prostacyclin analogues, it is important that these medications are continued to prevent rebound pulmonary hypertension and worsening symptoms [17,18]. In patients with a known PDA, clinicians should also maintain a high index of suspicion for endocarditis and septic emboli.

### 2.4. Pulmonic Stenosis

#### 2.4.1. Anatomy and Physiology

Pulmonic stenosis is defined as narrowing of the pulmonary valve which is located between the right ventricle and the main pulmonary artery. The majority of valvular stenosis cases are congenital and can occur in isolation or in association with other malformations [16]. Additionally pulmonic stenosis may be acquired, such as a downstream consequence of surgical reconstruction. Accordingly, the condition and its symptoms occur on a spectrum of severity. 

#### 2.4.2. Medical/Surgical Management

Indications for intervention in patients with pulmonic stenosis are based on the severity of stenosis and symptoms of heart failure which are otherwise unexplained. The severity of symptoms is generally based on the NYHA classification of cardiovascular disability as well as peak Doppler gradient across the stenotic valve. Treatment usually begins with balloon valvotomy but may require surgical intervention in some cases [5,19].

#### 2.4.3. Special Considerations

The probability of survival is similar to the general population if the patient has isolated valvular pulmonic stenosis. This can be greatly impacted if co-morbid conditions exist, or if the stenosis is the result of an alternative congenital or acquired disease state. When isolated (non-severe) pulmonic stenosis is present, the majority of patients are asymptomatic and do not require intervention [19]. ED presentations often center around post-surgical complications such as infection, arrhythmia, and heart failure.

### 2.5. Tetralogy of Fallot

#### 2.5.1. Anatomy and Physiology

Tetralogy of Fallot (ToF) consists of four anatomic features, which include right ventricular outflow tract (RVOT) obstruction, VSD, overriding aorta, and right ventricular (RV) hypertrophy. It is the most common form of congenital cyanotic heart disease. The degrees of pathophysiology of ToF are related to the degree of RVOT obstruction. Most patients will not demonstrate cyanosis at birth but will develop worsening cyanosis during the first few weeks and months of life [20].

#### 2.5.2. Medical/Surgical Management

Medical management may be required if there is severe right ventricular outflow tract obstruction initially. Treatment includes intravenous (IV) prostaglandin therapy to maintain ductal patency until surgical intervention is completed [5,21]. However, most patients with ToF undergo surgical repair, typically by one year of age, with the ideal age for optimum repair between three to six months [20,22]. Previously, ToF was repaired in two-staged procedures [22] with the initial stage involving the creation of a systemic-pulmonary shunt—Blalock–Thomas–Tausing shunt being the most common and classically involving anastomosis of the subclavian artery to the pulmonary artery. Primary intracardiac repair is now the mainstay treatment for most patients with ToF, with the main goals addressing relief of RVOT obstruction, separation of the pulmonary and systemic circulations, maintaining RV function, and minimizing pulmonary valvular disease [21,22]. The surgery includes patch closure of the VSD and enlarging the RVOT by correcting pulmonary stenosis, with an increasing emphasis in maintaining pulmonary valve competence [20,21,22].

#### 2.5.3. Special Considerations

Placement of a prior palliative shunt in these patients can affect blood pressure readings in their extremities as the subclavian arteries may be “sacrificed” during the initial palliative intervention. Adult patients with previously repaired ToF can develop long-term postoperative complications; commonly, pulmonary regurgitation, arrhythmias, and heart failure. With the continued advancement of surgical techniques repairing ToF, emergency clinicians must be aware of the cardiac sequelae that can occur in adult patients with ToF. ECG findings of the right bundle branch block and QRS durations >180 ms are not uncommon secondary to this anatomy; the wider the QRS, the higher the risk of ventricular arrhythmias and sudden cardiac death.

### 2.6. Coarctation

#### 2.6.1. Anatomy and Physiology

Aortic coarctation is a narrowing of the aorta, impeding distal flow and increasing vascular pressure proximal to the lesion. The most common concomitant congenital lesions include bicuspid aortic valve, VSD, valvular stenosis, berry aneurysms, and hypoplastic left heart, among others. Anatomical diagnosis may occur during prenatal sonography with progression to fetal echocardiography, or may occur postnatally, often after closure of the ductus arteriosus, which results in greater left ventricle afterload in combination with decreased perfusion distal to the lesion [23]. The result of the coarctation is classically a pressure gradient when comparing proximal and distal to the lesion and can be suspected when comparing upper and lower extremity blood pressure readings.

#### 2.6.2. Medical/Surgical Management

Medical management is typically initiated at the time of diagnosis. Evaluation, monitoring, and treatment of hypertension is common. Surgical management typically occurs prior to age two and definitive repairs include: end-to-end anastomosis, patch aortoplasty, and intra-aortic stenting, among others. The delay of surgical repair may lead to the formation of collateral blood flow, resistant hypertension, and increased mortality rates [23,24].

#### 2.6.3. Special Considerations

Adults with aortic coarctation manifest more cardiac morbidity than the general population, including earlier development of heart failure and longer hospitalizations for heart failure [25]. Patients with coarctation are at higher risk for aortic aneurysm [23]. Blood pressure readings post-repair are more accurate on the opposite side of the repair due to compromise of the subclavian artery during the surgery, which may lead to underestimation of the pressures.

### 2.7. Transposition of the Great Arteries (TGA)

#### 2.7.1. Anatomy and Physiology

Complete transposition of the great arteries (D-TGA), a condition in which the aorta arises from the right ventricle (RV) and the pulmonary artery (PA) from the left ventricle (LV), is incompatible with life without a co-existing shunt [26]. Cases present when the ductus arteriosus closes or during childhood. In congenitally-corrected TGA (ccTGA or L-TGA), the patient is born with the ventricles reversed; diagnosis in adulthood is common and depends on associated lesions such as ventricular septal defects, tricuspid valve abnormalities, pulmonary stenosis, and heart block [26].

#### 2.7.2. Medical/Surgical Management

**Figure 1:**
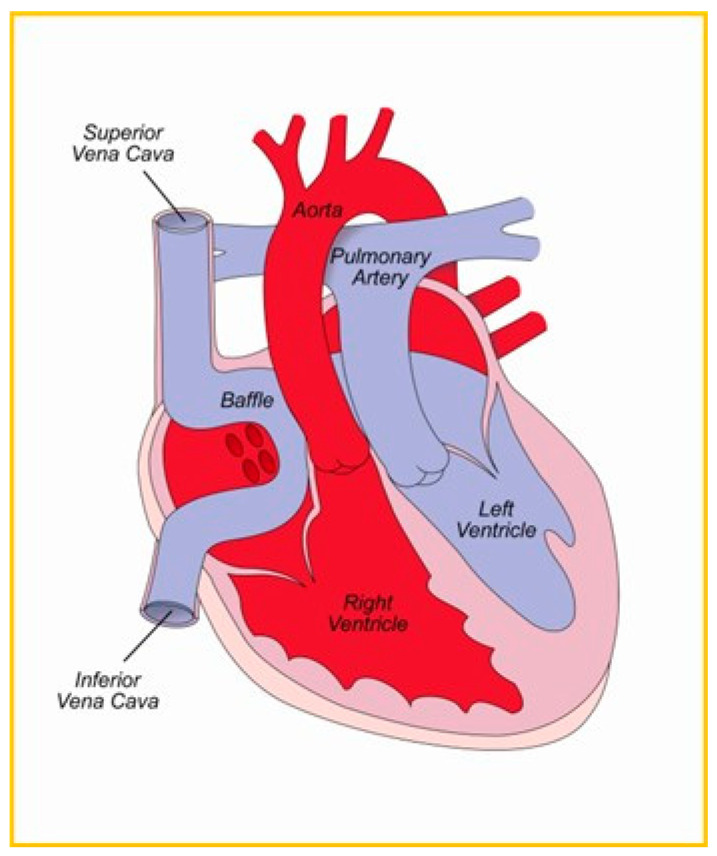
Atrial switch: DTGA s/p atrial switch and LTGA have systemic RV pumping to aorta.

D-TGA management at birth involves prostaglandin E1 and balloon atrial septostomy, then surgical palliation [27]. Before the 1980s, the atrial switch created interatrial baffles redirecting oxygenated blood through the RV and out the aorta [26,28]. Arterial switch now predominates, resulting in the LV serving the systemic circulation (Figure 1).

**Figure 2:**
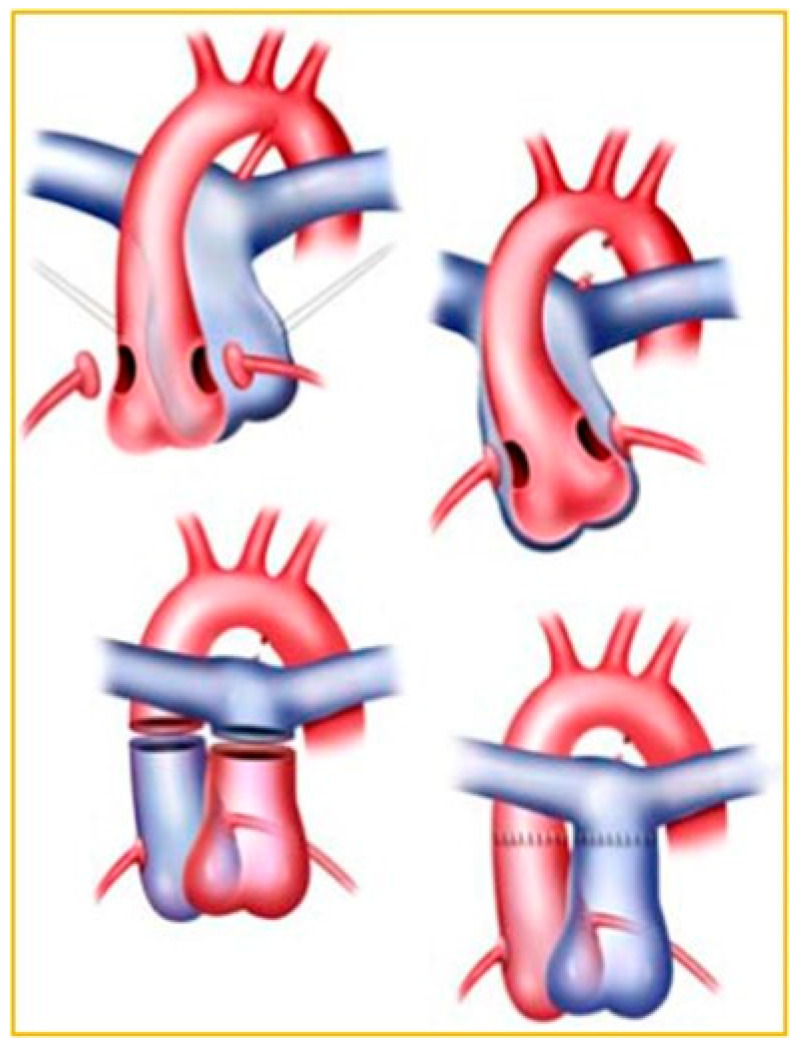
Arterial switch: D-DTGA s/p arterial switch, LV is systemic ventricle.

D-TGA s/p atrial switch and LTGA have systemic RV which is perfused by the right coronary artery. D-TGA s/p arterial switch (Figure 2) has a systemic left ventricle 

#### 2.7.3. Special Considerations

Emergency presentations of TGA/ccTGA vary with repair. Atrial switch patients experience RV failure from maintaining systemic circulation and also experience a high rate of atrial arrhythmias; rhythm control is preferred [28,29]. Additional complications include systemic tricuspid regurgitation, pulmonary hypertension, baffle leak (with paradoxical embolism and stroke), and baffle stenosis/obstruction (symptoms of SVC or IVC stenosis) [28,29]. In arterial switch patients, reattached coronary arteries may become narrowed and cause ischemia; neo-aortic dilation/regurgitation and PA stenosis may also occur [28]. Ischemia may be painless due to denervation [30]. Stress testing and tomographic imaging of the coronaries are options for ischemic evaluation in patients experiencing chest pain [26].

### 2.8. Aortic Stenosis

#### 2.8.1. Anatomy and Physiology

Aortic stenosis (AS) is defined as narrowing of the aortic valve resulting in outflow obstruction and progressive alteration of hemodynamics. In most individuals, the aortic valve consists of three leaflets (tricuspid aortic valve). Atherosclerotic processes (endothelial injury and inflammation, lipid infiltration, calcium depositions, and ossification) contribute to the gradual development of calcific valvular disease, and progression of AS in individuals with tricuspid aortic valves and the often earlier manifestation of symptoms in patients with underlying bicuspid aortic valves [31]. 

#### 2.8.2. Medical/Surgical Management

Clinical decision-making is based on several indices, including velocity gradient across the valve, mean pressure gradient, and valve area [32]. The primary therapy for AS is surgical, consisting of either open aortic valve replacement (AVR) or transcatheter aortic valve replacement (TAVR). These two interventions have been effective in reducing symptoms from aortic stenosis and extending life expectancy [31]. Valvuloplasty is a therapeutic option in younger patients without significant aortic insufficiency, as well in patients who are poor surgical candidates [33]. In these individuals, the probable need of reintervention or AVR is high [33].

Medical management can result in improved hemodynamic indices; however, it does not improve symptoms or life expectancy [31]. Its role in managing patients with AS is therefore limited. Medical management consists of prevention of progression of atherosclerotic disease, antihypertensive therapy, and limitation of physical activity. These recommendations are considered in patients because of their preference or who are poor surgical candidates or as bridge to operative treatment.

#### 2.8.3. Special Considerations

The natural history of AS is characterized by a prolonged asymptomatic period followed by a progression of symptoms, often characterized initially as exercise intolerance. Classic ED presentations of aortic stenosis include syncope, angina, heart failure, dysrhythmia, and sudden cardiac death [31,33].

### 2.9. Univentricular Heart

#### 2.9.1. Anatomy and Physiology

Univentricular heart is a heterogeneous group of complex congenital heart disease where there is one dominant and one hypoplastic ventricle or atrioventricular malformations and septal abnormalities that preclude biventricular repair. Subtypes of this abnormality include hypoplastic left heart syndrome, atrioventricular valve atresia, and double-inlet left ventricle. Patients typically present early in life with either cyanosis, heart failure, or failure to thrive.

#### 2.9.2. Medical/Surgical Management

Many will have multiple-staged palliative procedures such as systemic-pulmonary shunts, atrial septostomy, or pulmonary artery banding. The final palliative surgical intervention culminates with the Fontan operation where all the systemic venous return is routed to the pulmonary artery without a pump while thr dominant ventricle serves as the systemic pump. The Fontan surgery has many variations from the classic Fontan where the right atrial appendage is anastomosed to the pulmonary circuit to the extracardiac Fontan where a conduit is used to route vena caval flow to the pulmonary artery. Success of the Fontan operation depends on having a preserved systemic ventricular systolic function, normal pulmonary and left atrial pressures, minimal valvular abnormalities, patent pulmonary arteries, and sinus rhythm.

As the Fontan is a palliative procedure, patients require long-term multidisciplinary care as there are a multitude of sequelae such as progressive ventricular systolic and diastolic dysfunction, arrhythmias, thromboembolism, hepatic impairment, cyanosis, protein losing enteropathy, and the need for re-intervention. Atrial arrhythmias are prevalent and their onset can herald any underlying hemodynamic abnormality. Patients who are post-Fontan operation presenting with atrial arrhythmias can develop heart failure and resuming normal sinus rhythm is important to maintain the atrial kick contribution to their cardiac output. Rate control would be the initial approach in their care but consultation with cardiologists with expertise in congenital heart disease as well as electrophysiologists would be vital in their management and discussion on the best options to regain sinus rhythm which can include anticoagulation, anti-arrhythmics, and transesophageal echocardiography-guided cardioversion.

#### 2.9.3. Special Considerations

Diuresis can be initiated in the setting of a pulmonary edema and fluid overload, although it should be performed cautiously as the Fontan circulation is preload-dependent. Care should be taken with sedation in the patients post-Fontan given sensitive hemodynamics and involving cardiac anesthesia would be prudent if available.

## 3. Critical Considerations of Emergency Department Presentation

Overall, cardiovascular reasons account for approximately 77% of all deaths in patients with adult CHD, with approximately half attributable to chronic heart failure [5]. The most common emergency in adult patients with CHD is arrhythmia, accounting for 37% of emergency admissions [34]. Other common presentations include dyspnea, chest pain, and infection. These patients warrant consideration of the same disease etiologies that all ED patients undergo, as well as a host of issues specific to their underlying heart diseases. 

### 3.1. Arrhythmia

The prevalence of arrhythmias in patients with CHD is a cause of significant morbidity and mortality. With advances in therapy for CHD, the incidence of arrhythmias in adult patients with a history of CHD is high. For instance, approximately half of adult patients experience atrial arrhythmias by the age of 65 [35]. The etiology of these arrhythmias is secondary to either the intrinsic structural abnormality of the CHD or scarring from operative repair of the condition. Advances in anatomic mapping and surgical techniques over the past several decades have both decreased arrhythmia burden and increased life expectancy in patients with CHD. 

#### 3.1.1. Tachydysrhythmias

Tachydysrhythmias can be categorized as accessory pathways, twin AV nodes, intra-arterial reentrant tachycardia (IART), atrial fibrillation, and ventricular tachycardia [36]. Accessory pathways are typically associated with Ebstein anomaly, as well as levo-transposition of the great arteries (L-TGA). Wolff–Parkinson–White syndrome may coexist in patients with Ebstein anomaly of the tricuspid valve in 20% of cases [36]. Intra-atrial re-entrant tachycardia is frequently the result of corrective operative procedures such as Mustard, Senning, and Fontan, as well as the repair of atrial septal defects. These procedures cause myocardial stress on the right atrium, resulting in IART. Atrial fibrillation is usually secondary to hemodynamic abnormalities involving the left atrium, such as mitral valve disease, aortic stenosis, and unrepaired single ventricle disorders.

The risk for ventricular tachycardias (VT) increases after the second decade of life in patients with CHD. Congenital heart defects associated with VT include aortic valve disease, L-TGA, Ebstein anomaly, congenital single ventricle, Eisenmenger’s syndrome, and Tetralogy of Fallot (ToF). The risk for sudden cardiac death increases by 2% per decade in patients with ToF, primarily due to VT [36].

#### 3.1.2. Bradydysrhythmias

Bradydysrhythmias also occur frequently in CHD patients, resulting directly from the congenital malformations themselves and also indirectly from the repairs. 

#### 3.1.3. Evaluation and Treatment

The typical presentation of patients with CHD who experience arrhythmias include dizziness, palpitations, syncope, dyspnea, chest pain, and sudden cardiac arrest. A thorough history regarding a patient’s specific congenital heart defect and subsequent surgical history is essential. Additionally, careful evaluation using ECG, telemetry, laboratory studies, and imaging studies are of essence in the ED. Cardiology and electrophysiology evaluation with expertise in CHD is essential in further diagnostic testing with Holter monitoring, echocardiogram, and cardiac mapping. The treatment of IART includes antiarrhythmic medications, catheter ablation, pacemaker implantation, or surgical intervention with a modified maze procedure. Atrial fibrillation is treated with rate and rhythm control with medication, cardioversion, as well as anticoagulation to prevent thromboembolic events. In patients with SA node dysfunction who are symptomatic, pacemaker insertion is the mainstay of therapy. Indications for the implantation of ICD in patients with CHD continues to evolve. Typically, in patients with ToF, ventricular outflow obstructions and transposition of the great arteries are candidates for ICD [36]. In an emergent setting, standard ACLS pathways apply. 

### 3.2. Shortness of Breath

Acute dyspnea is a frequent ED presentation in adult patients with congenital heart disease. The development of heart failure affects 20–50% of this population and is the main cause of death [16]. The etiology of heart failure in these patients may be left ventricular systolic dysfunction/heart failure with reduced ejection fracture (HFrEF), left ventricular diastolic dysfunction/heart failure with preserved ejection fraction (HFpEF), or right heart failure (RHF). Arrhythmias and valvular abnormalities may contribute to the development of heart failure. ED clinicians should be able to quickly differentiate between the causes of heart failure as management strategies may change based on etiology. 

Point-of-care ultrasound (POCUS) findings consistent with HFrEF include moderate-severely reduced EF; quantitative measurements of LV function are generally not required in the acute critical setting. Conversely, findings of left ventricular hypertrophy and left atrial enlargement should prompt clinicians to consider the possibility of diastolic dysfunction seen in HFpEF. 

In adult patients with CHD, it is important to maintain a high index of suspicion for PH and RHF as a cause of symptoms. PH causes right ventricular hypertrophy, followed by dilation and dysfunction. There are only a few studies on the assessment and treatment of PH in the ED [17]; however, it is critical to recognize PH/RV failure in critically ill patients because this will complicate management. These patients are frequently fluid unresponsive and require early vasopressor support [17]. POCUS findings consistent with RV dysfunction include dilation defined at a 1:1 RV to LV ratio (or RV basal diameter greater than 42 mm), and RV hypertrophy with a free wall thickness greater than 5 mm [17]. 

Management of adult patients with a history of CHD presenting with dyspnea presumed to be the result of pulmonary hypertension or heart failure can be complex. Initial emergency room care should be directed to the primary survey and includes an assessment of pulse oximetry and vital signs [37]. The treatment of underlying arrhythmia or ischemia can often immediately improve dyspnea. While long-term oxygen therapy may not be beneficial for CHD patients, oxygen therapy in the acute setting is likely useful and will improve symptoms for CHD patients in distress [5].

CHD patients are at higher risk of pulmonary artery thrombosis as the result of ventricular dysfunction or slow pulmonary artery blood flow stemming from structural or functional changes in vasculature. These cases require immediate anticoagulation. In cases of extremis, such as massive thrombus resulting in severe hypoxemia or hypotension, thrombolysis should be considered.

If the patient is exhibiting signs of volume overload, including pulmonary and peripheral edema, diuretics and vasodilators may be considered in the ED setting. Adult CHD patients presenting with cardiogenic shock should follow similar treatment patterns to the general population, with an emphasis on airway, breathing, and circulation. Early stabilization and transport to a tertiary care center with CHD specialization should be considered [34].

### 3.3. Chest Pain

#### 3.3.1. Acute Coronary Syndrome (ACS)

Many patients with adult CHD experience an increased risk of coronary artery disease (CAD). This is likely multifactorial, from the defect itself, surgical repairs, and increased prevalence of risk factors such as obesity, hypertension, and sedentary lifestyles in several CHD subtypes [30]. Surgeries where coronary arteries are reattached may lead to coronary stenosis and non-atherosclerotic ischemic events [28]. In patients with unrepaired right-to-left shunts, paradoxical embolism may lead to embolic myocardial infarction [38]. Although patients may present with chest pain, emergency physicians should remain alert to ACS in atypical presentations due to denervations that occur during the surgical repair of CHD [30].

#### 3.3.2. Pulmonary Embolism (PE)

Although studies are limited examining PE rates, adult CHD patients may exist in a pro-thrombotic state due to chronic hypoxia and resultant polycythemia [39]. Patients also undergo operations which put them at risk of developing PE in the post-surgical period. Although PE remains rare in the pediatric population, adult CHD patients with severe disease and dilated cardiomyopathy may develop intracardiac thrombi and arrhythmias which put them at risk of developing PE [40].

#### 3.3.3. Aortic Dissection

The likelihood of aortic dissection is variable based on the nature of the congenital lesion and prior operative interventions. These factors work to lower the typical age at which dissection may present. Younger patients who have CHD are more likely to develop aortic dissection than individuals with normal anatomy [41]. As individuals with CHD age, they are susceptible to the accumulated additional stresses of typical cardiac disease risk factors, including hypertension, hyperlipidemia, diabetes, etc. There are also vascular histological abnormalities associated with many types of CHD, such as the association of Erdheim cystic medial necrosis with bicuspid aortic valve and loss of elasticity with ToF. Acquired abnormalities of the vessel wall are associated with surgical corrections such as that performed for TGA [42]. Complications from correction of coarctation of the aorta may include dilation of the proximal vessel. Similarly, ToF (with or without repair) is a risk factor for aortic root dilation, demonstrated to affect 15–51% of adults, though progression to aortic dissection in ToF is uncommon [42,43].

The general incidence of aortic dissection in the setting of CHD is not definitively known but suspected to be low. The most important gross anatomic risk factor is bicuspid aortic valve [44]. Individuals with CHD and chest pain, displaying other concerning signs of aortic syndrome, should have an evaluation for this regardless of age.

#### 3.3.4. Other Chest Pain

Pulmonary hypertension may present with acute chest pain, in addition to shortness of breath which may be related to extrinsic compression of the left coronary artery from an enlarged pulmonary artery. This is explored more in depth (previously/subsequently). Studies evaluating diverse causes of chest pain in adults with CHD have found that idiopathic pain is common. Additional considerations include pulmonary hemorrhage, arrhythmia, musculoskeletal, and other non-cardiovascular causes [45].

### 3.4. Infection

Adults with CHD represent a unique population that requires specialized care when presenting to the ED. This group of patients is at increased risk of infection, particularly infective endocarditis, and many have concomitant immunocompromise. Infective endocarditis occurs at a rate of 3 to 7 per 100,000 person-years in the general population, but in the adult CHD population, the incidence is roughly 1.1 per 1000 patient-years [46,47]. Conditions that increase the risk of infective endocarditis include prosthetic valves, prosthetic rings, previous infective endocarditis, cyanotic CHD, and any CHD repaired within the preceding six months [34]. In addition to infective endocarditis, patients with cyanotic CHD are at higher risk for pneumonia and brain abscesses [48].

Patients with infectious complications of CHD require a multidisciplinary approach. Early consultation with cardiology and infectious disease specialists is indicated. In some cases, surgical intervention may be necessary, and cardiac surgical consultation can help with risk–benefit analysis prior to surgery.

### 3.5. Cyanotic Heart Disease

A number of common congenital heart defects can result in cyanosis, such as through right to left shunting as in ES. ECGs demonstrate right axis deviation with RA enlargement, RV hypertrophy, and RV strain. Patients may have severe hypoxemia and cyanosis without distress. These patients often do well until their 20s, with mortality increasing from 25% at age 30 to 45% at age 50 [49]. Deaths are most often due to sudden cardiac events, congestive heart failure, hemoptysis, infection, and pregnancy. Due to their chronic hypoxemia, these patients often have elevated hematocrits and may develop issue with hyperviscosity such as headaches, dizziness, myalgias, and visual changes, though rarely until the hematocrit exceeds 65%. In some cases, these symptoms can be relieved by withdrawing up to 500 cc of blood and infusing an equal amount of isotonic fluids, though regular use of this practice is discouraged due to the patients’ impaired oxygen-carrying capacity and increased risk of stroke from their shunts. Any new headache warrants cerebral imaging as the patients are at high risk for abscess, hemorrhage, and stroke. Of note, IV tubing with filters should always be used for these and other infusions given the patients’ right to left shunts, which are a risk factor for stroke and other complications [5]. Patients with Eisenmenger syndrome are at risk of thrombosis as well as bleeding due to hemostatic abnormalities. They are at risk of hemoptysis due to bleeding diasthesis, bronchitis, rupture of aortopulmonary collaterals, or pulmonary artery injury. Procedures that involve sedation such as bronchoscopy should be avoided as they can cause clinical decompensation with a reduction in the systemic vascular resistance. Therapy for hemoptysis would vary depending on the etiology and can include supplementary oxygen, antibiotics, infusing blood products, or embolization of the culprit bleeding vessel.

## 4. Conclusions

Overall, adult patients with congenital heart disease represent a complex and diverse group of patients who are increasingly prevalent within the ED. Emergency physicians must be aware of the myriad of clinical ways these patients may present, especially in the areas that they diverge from patients without their cardiac malformations. As noted above, not every CHD patient has the same anatomy nor faces the same risks as an adult, so clinicians need to have some knowledge of the anatomy, physiology, and complications of these relatively common conditions in order to take appropriate care of these patients.

## Data Availability

Not applicable.
